# New frontiers in type I diabetes treatment: the impact of mesenchymal stromal cells on long-term complications

**DOI:** 10.3389/fcdhc.2025.1586061

**Published:** 2025-05-19

**Authors:** Deeptha Bejugam, Sarah Bu, Athena N. Nguyen, Mariam Yaltaghian, Kinga K Smolen

**Affiliations:** ^1^ Georgetown University School of Medicine, Washington, DC, United States; ^2^ Precision Vaccines Program, Department of Pediatrics, Boston Children’s Hospital, Boston, MA, United States; ^3^ Harvard Medical School, Boston, MA, United States

**Keywords:** type I diabetes, stem cells, diabetic nephropathy, diabetic wound healing, diabetic cardiovascular disease, mesenchymal stromal cells

## Abstract

Type 1 diabetes (T1D) is not only a disorder of insulin production from beta cell destruction, but also a progressive condition that brings about life-threatening complications such as diabetic nephropathy, impaired wound recovery, and cardiovascular disease. Mesenchymal stromal cell (MSC) use has recently become an encouraging new way to treat these complications and can result in better health outcomes for T1D patients. Some research has shown that MSC injections into mice and rat models have resulted in reduced mesangial cell thickening, inflammatory mediator recruitment, proteinuria, and fibrosis normally seen in diabetic nephropathy. Other studies have demonstrated that MSCs aid wound healing by increasing anti-inflammatory M2 macrophage differentiation, stimulating angiogenesis and collagen synthesis, and signaling the proliferation and migration of dermal fibroblasts toward injury sites. Additionally, there is evidence that MSCs are capable of activating the PI3K pathway and exhibiting antioxidant effects in murine models experiencing diabetic-related heart disease. However, given these efforts, further research is needed to establish the prolonged safety and efficacy of MSC use in humans to treat T1D.

## 1 Introduction

Type 1 Diabetes (T1D), a chronic disease affecting an estimated 2 million Americans, is characterized by the autoimmune destruction of the body’s pancreatic beta cells (1). This results in a loss of endogenous insulin production, leading to insulin deficiency and dysregulation of blood glucose levels with potentially fatal consequences (2). Current treatments for T1D include replacement therapy of insulin in the form of injections or a pump, regular blood glucose monitoring, dietary control, and exercise regimens aimed at preventing both acute and chronic health complications (3). Despite these treatment options, complications such as cardiovascular disease, nephropathy, and poorly healing cutaneous wounds, amongst others, can still occur with inadequate management (4–7).

**Figure 1:**
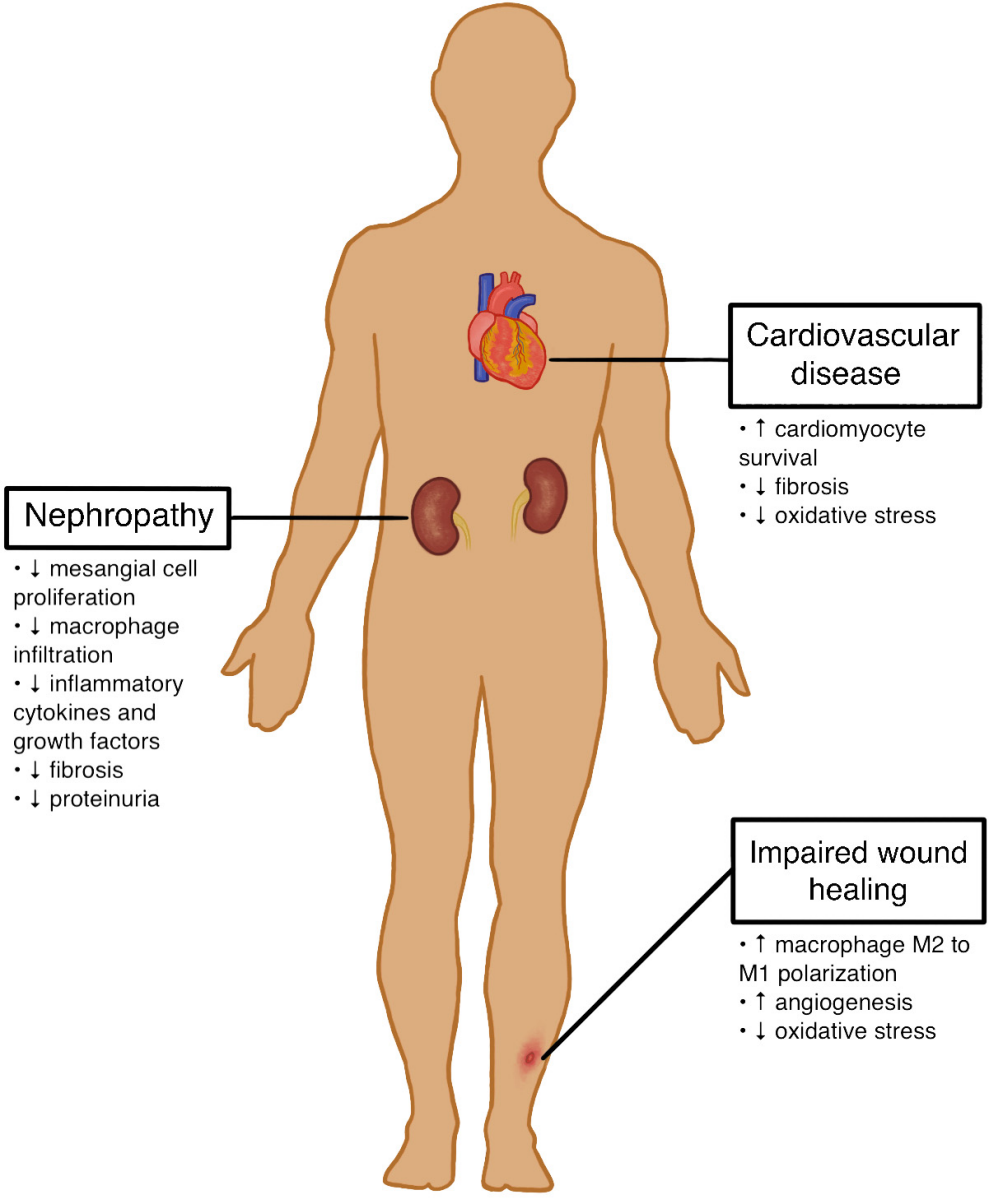
Potential Effects of MSC Therapy on T1D Complications.

**Figure 2:**
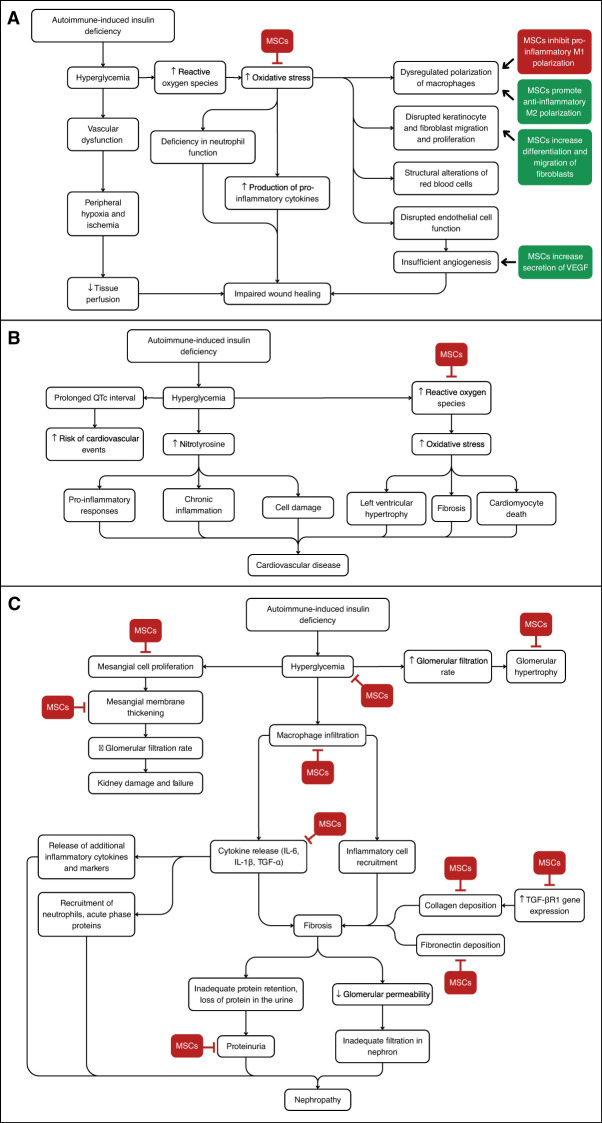
Overview of the Signaling Pathways Discussed and Key Points Where MSC Therapy May Intervene. **(A)** Development of diabetic impaired wound healing. MSC therapy can help decrease oxidative stress and inhibit macrophage M1 polarization, while increasing macrophage M2 polarization and fibroblast differentiation. **(B)** Development of diabetic cardiovascular disease. MSC therapy may decrease oxidative stress. **(C)** Development of diabetic nephropathy. MSC therapy can inhibit several steps including mesangial cell proliferation, collagen deposition, and inflammatory cytokine release, among others.

Given the persistent life-threatening complications in patients with T1D, developing alternative therapies is an important next step toward improving diabetic care. Mesenchymal stromal cells (MSCs) have recently become a promising novel treatment for complications related to T1D. MSCs are defined as multipotent cells with the ability to differentiate into a variety of mesenchymal cell lineages such as adipocytes, osteoblasts, and chondroblasts *in vitro* (8). Human MSCs are capable of differentiating into insulin-producing cells, protecting engineered pancreatic islet beta cells from hypoxia, and promoting the regeneration of pancreatic islet beta cells (9–13). These characteristics highlight the potential of these cells in regenerative medicine, particularly for treating various T1D complications such as cardiovascular disease, nephropathy, and impaired wound healing. The potential effects of MSC therapy on these T1D complications are outlined in **Figures 1** and **2**.

The use of MSCs presents an important opportunity for the future of T1D treatments. Although further research is needed to determine the long-term safety and efficacy of MSC therapies, this review aims to analyze the current evidence and provide insights for future research and clinical applications. We will delve into the effects of MSCs on the T1D complications of diabetic nephropathy, impaired wound healing, and cardiovascular disease. We will also address important challenges surrounding MSC use to treat T1D in humans and why, despite these challenges, MSC therapy is still a worthwhile treatment avenue to further explore.

### 1.1 MSC use in diabetic nephropathy

T1D can lead to hemodynamic and metabolic dysregulation, which can progress to chronic renal failure. MSC therapy has been a promising avenue in several pre-clinical *in vivo* and *in vitro* studies to repair nephron damage and other profound effects of diabetic nephropathy (4–7). **Table 1** shows results from MSC therapy in diabetic mice and rat models.

**Table 1:** Mesenchymal stromal cell therapy in mouse and rat models demonstrating the effect on diabetic nephropathy, diabetic wound healing, and cardiovascular disease.

Complication Addressed	Type of Mesenchymal Stromal Cell	Model	Dosage	Effect on Function and Structure	Reference
Diabetic Nephropathy	Multipotent Stromal Cell from Human Bone Marrow	Mice	2.5*10^6^ Multipotent Stromal Cell injected on day 10 and 17	Decrease in mesangial thickening -Decrease in macrophage infiltration -Significantly lowered blood glucose compared to untreated controls on day 32	(14)
	Human Adipose-derived Stem cells	Sprague-Dawley rats injected in the tail vein	5 times of 5*10^6^ of Human Adipose-derived Stem Cells at 4 weekly intervals	-Reduction in proteinuria after 24 weeks -Reduction in glomerular hypertrophy and interstitial injury -Decrease of WT-1 and synaptopodin expression	(15)
	Human Umbilical cord-derived mesenchymal Stem cells	Sprague-Dawley rats injected in the tail vein	Single dose of 5*10^5^	-Decrease in mesangial thickening -Decrease in proteinuria -Decrease of collagen accumulation in diabetic kidneys -Stem cells were engrafted in diabetic kidneys and remained in kidneys for 4 weeks	(27)
	Human Umbilical cord-derived mesenchymal Stem Cells	Sprague-Dawley rats injected in the tail vein	Single dose of 1*10^6^ Stem Cells at 4 weeks after onset of diabetes	-Decreased proteinuria -Decreased fibronectin -Decreased renal E-cadherin -Increase in α-SMA -Stem cells were engrafted in diabetic kidneys	(24)
	Human Umbilical cord-derived mesenchymal Stem Cells	Sprague-Dawley rats injected in the tail vein	2*10^6^ Stem Cells at 6 weeks	At two weeks after injection: -Decrease in renal fibrosis, vacuole degeneration, inflammatory cell infiltrate, and interstitial fibrosis -Decrease in Il-6, IL-1β, and TNF-α -Increase epidermal growth factor, fibroblast growth factor, and vascular endothelial growth factor	(16)
	Human bone marrow-derived mesenchymal stem cells, engineered to overexpress miRNA-let7c (miR-let7c-MSCs)	Male C57BL/6J mice	1 × 10^6^ miR-let7c MSCs intravenously	-Attenuates TGF-β1-driven TGF-βR1 gene expression -Decrease in interstitial collagen -Anti-fibrotic effects (2)	(17)
Diabetic Wound Healing	Melatonin-stimulated exosomes from human bone marrow-derived mesenchymal stem cells	Sprague-Dawley rats via intraperitoneal injection of STZ	One multisite subcutaneous injection of unspecified dose	-Decreased size of diabetic wounds -Improved angiogenesis and collagen synthesis -Strengthened anti-inflammatory effect of M2 macrophages and weakened pro-inflammatory effect of M1 macrophages	(18)
	Hypoxic Human adipose-derived stem cells exosomes	BALB/c nude mice	One dose of 2 mg in 100 μL PBS subcutaneously injected	-Improved wound closure and faster wound closure rates -Skin wounds demonstrated complete re-epithelialization and cuticle covering on the epidermis -Higher expression of TGF-β, COLI, PDGF and VEGF -Increased fibroblast migration for remodeling and production of extracellular matrix proteins	(36)
	Human umbilical cord mesenchymal Stem cell-derived exosomes	Male C57BL/6J mice	Doses of 100 µl of PBS, 50 μg/ml of UMSCs-Exos, and 100 μg/ml of UMSCs-Exos locally injected on days 0, 3, 5, 7, 9, and 11	-Improved rate of wound healing, greater granulation tissue formation -Promoted angiogenesis and reduced oxidative stress -Lower levels of inflammatory mediators IL-1β, IL-6, and TNF-α	(19)
	Transgenic adult mice bone-marrow derived mesenchymal stem cells	Female mice homozygous for the Leprdb mutation	One dose of 1 × 106 MSCs injected intradermally	-Improved wound closure and decreased wound size -Increased collagen production -Decreased MMP-9 gene expression and decreased proteolysis	(20)
	Human umbilical cord derived stem cells	Humans with chronic non-healing ulcers, including diabetic ulcers	2 × 107 MSCs injected subcutaneously and intramuscularly	-Reduction in wound size and increased wound healing rate -Growth and maturation of granulation tissue –Increased blood perfusion and increased transcutaneous oxygen pressure	(21)
	Allogeneic human adipose-derived mesenchymal stem cells	Humans with chronic diabetic foot ulcers	Mean of 6 × 106 cells injected intradermally	Decreased time to wound closure and increased physical functioning	(22)
Diabetic Cardiovascular Disease	Human adipose-derived mesenchymal stem cells-derived extracellular vesicles (EVs)	Dental follicle cells of xenogeneic bioengineered bio-roots	Incubation with PKH26-marked EVs for time frames of 3, 6, 12, 24, and 48 h at 37°C followed by PBS washing	-Reduced cell apoptosis, reactive oxygen species generation, mitochondrial changes, and DNA damage -Activation of the phosphatidylinositol 3-kinase (PI3K)/Akt pathway	(23)

One such complication of diabetic nephropathy is mesangial cell proliferation caused by hyperglycemia. Mesangial cells are involved in several renal functions including phagocytosis and cell-to-cell signaling, and the proliferation of these cells has been a contributing factor to chronic renal failure in T1D. MSC therapy addresses these renal complications in mice and rat models with significant reductions in mesangial cell proliferation. For instance, *one study* showed that multipotent stromal cells from human bone marrow in mice led to significantly lowered blood glucose after 32 days compared to untreated controls. Simultaneously, there was also the appearance of human cells that differentiated into glomeruli and a decrease in mesangial thickening (14). Similarly, when human umbilical cord blood-derived mesenchymal were engrafted in the kidneys of Sprague-Dawley rats, mesangial membrane thickening was reduced).

MSC therapy may not only attenuate mesangial cell proliferation, but it may also decrease inflammation from inflammatory cells and signaling molecules that exacerbate nephron damage. MSCs derived from human bone marrow injected into mice showed significantly lower macrophage infiltration. Since macrophages typically release cytokines that recruit inflammatory cells to the nephron, which may increase fibrosis and scarring, lower macrophage infiltration can consequently mitigate the damage caused by inflammation of the kidneys (14). Human umbilical cord-derived MSCs decreased levels of interleukin (IL)-6, IL-1β, and transforming growth factor (TGF)-α (16). IL-6, IL-1β, and TGF-α are pro-inflammatory cytokines involved in both acute and chronic inflammation and are actors that recruit inflammatory cells and mediators such as neutrophils, acute phase proteins, and release of other inflammatory cytokines and markers (16). Thus, a reduction in these cytokines may prevent the damaging effects of inflammation of the nephron in diabetic nephropathy by decreasing the recruitment of mediators that promote hyper-inflammation.

The degree of renal fibrosis also contributes to the progression of chronic renal failure as a greater degree of fibrosis decreases the permeability of the glomerulus, resulting in inadequate filtration from the nephron. In examining the effects of MSCs on fibrosis, delayed administration of human umbilical cord blood-derived MSCs attenuated the progression of kidney injury by decreasing deposition of collagen, a structural extracellular matrix protein, and fibronectin, a recruiter of extracellular matrix components (24). Likewise, when male C57BL/6J mice engineered to overexpress miRNA-let7c were injected with human bone marrow-derived MSCs, TGF-β1-driven TGF-βR1 gene expression, which significantly increased collagen expression, was attenuated (17). Subsequently, the deposition of extracellular matrix components contributes to fibrosis, and by attenuating the deposition of these components, MSCs may lead to anti-fibrotic effects, mitigating the filtration impairment mediated by T1D.

A marker for glomerular damage caused by diabetic nephropathy is proteinuria, as fibrosis and nephron damage lead to inadequate retention of proteins in the blood and into the urine. Human adipose-derived MSCs injected into Sprague-Dawley rats led to significantly decreased proteinuria after 24 weeks of injection, while reducing glomerular hypertrophy (15). Moreover, human umbilical cord-derived MSCs injected into Sprague-Dawley rats also led to marked reductions in proteinuria (24). Since proteinuria is a marker for kidney damage from fibrosis and glomerular damage, MSC injections may show promise in reversing the nephrogenic effects of fibrosis and glomerular damage in diabetic nephropathy (25, 26).

The injection of these MSCs was performed in animal models. Additional research is needed to show the translational effects in humans in a safe and efficacious manner. However, the injection of MSCs from different lineages shows promise in reducing mesangial cell thickening, inflammatory mediator recruitment, fibrosis, and proteinuria seen in diabetic nephropathy that contribute to chronic renal failure (14–17, 24–27).

### 1.2 MSC use in diabetic wound healing

For T1D patients, cutaneous diabetic wounds, such as diabetic foot ulcers, are serious complications that can arise from diabetes with neuropathic abnormalities or diabetes with peripheral artery disease of the lower extremities (28, 29). Wounds in diabetic patients can progress more rapidly due to certain risk factors including vascular dysfunction with peripheral hypoxia and ischemia, chronic inflammation, peripheral neuropathy, and hyperglycemia (30, 31). Barriers and dysfunction in the healing of diabetic wounds are commonly attributed to poor glycemic control and the resulting hyperglycemia in patients. Hyperglycemic states and their associated states of oxidative stress lead to dysregulated polarization and modulation of cells, such as macrophages, neutrophils, keratinocytes, and endothelial cells, that otherwise promote wound healing (31). Deficiencies in neutrophil function, keratinocyte and fibroblast migration and proliferation, continuous production of pro-inflammatory cytokines, and structural alterations to red blood cells all contribute to delays in wound healing (31). Currently, treatments for diabetic wounds include debridement supplemented with pharmacological therapy, using growth factors and skin substitutes, and applying negative pressure wound therapy (28, 29). However, the use of MSCs in treating diabetic wounds holds promise as it can improve nearly all stages of wound healing between inflammation regulation, angiogenesis, and tissue remodeling (32–34).

One of the benefits of MSCs in modulating inflammatory processes to improve diabetic wound healing involves their ability to polarize classically activated, pro-inflammatory macrophages known as M1 macrophages to M2 macrophages which are conditionally activated and possess anti-inflammatory properties. In diabetic rat models, melatonin-stimulated exosomes from bone-marrow derived MSCs increased the ratio of M2 to M1 polarization of macrophages through respective promotion and inhibition with increases in the M2 macrophages, leading to shortening of the inflammatory periods that hinder healing for diabetic wounds (18). M2 macrophage-driven decreases in inflammation additionally encourage improvements in subsequent wound healing stages by promoting angiogenesis as well as collagen synthesis involved in tissue remodeling (18). The anti-inflammatory mechanisms of MSCs allow for amelioration of diabetic wounds across several stages of healing and prevent further diabetic wound pathogenesis (18, 32–35).

MSCs have been reported to improve angiogenesis of diabetic wounds by reducing oxidative stress and eliciting secretion of associated growth factors. Hypoxic exosomes from adipose-derived MSCs transplanted into diabetic wound nude mouse models found that transplanted MSCs could accelerate high-quality healing of diabetic wounds by secreting higher levels of vascular endothelial growth factor (VEGF) (36). Higher levels of VEGF stimulated differentiation of progenitor cells into endothelial cells and extracellular matrix progenitors as well as angiogenesis through the PI3K/threonine kinase signaling pathway, overall increasing synthesis of capillaries, granulation tissues, and epithelium (36). MSCs promote environments conducive to angiogenesis by regulating oxidative stress damage occurring in diabetic cutaneous wounds (19). Administration of human umbilical cord MSCs in mouse models with diabetic cutaneous wounds found that exosomes derived from the administered cells accelerate diabetic cutaneous wound healing via amelioration of oxidative stress and enhancement of angiogenesis, counteracting the pathogenic effects of hyperglycemia on chronic wounds (19).

Further effects of MSCs in diabetic wound healing have been demonstrated with their capabilities in cell differentiation, cell signaling, and their regulation of key enzymes to improve tissue remodeling. In addition to studies showing the use of human bone-marrow derived MSCs in promoting angiogenesis and possessing the ability to differentiate in keratinocytes and endotheliocytes responsible for tissue remodeling, adipose MSCs transplanted into diabetic wound nude mice models signaled for early proliferation and dermal fibroblast migration towards remodeling (18, 19, 37). MSCs within *in vivo* murine diabetic wound healing models have been shown to correct dysregulation of matrix metalloproteinase-9 levels in impaired diabetic wound healing where dermal extracellular matrix protein profiles favor proteolysis and further pathogenesis (20).

Animal models further demonstrate how MSCs could behave synergistically to improve diabetic wounds, such as the action of MSCs on the PI3K/threonine kinase signaling pathway. In addition to MSC-derived exosomes promoting angiogenesis through this pathway, the PI3K/threonine kinase pathway is upregulated by MSCs through an upstream PTEN enzyme target to suppress inflammation and remediate diabetic wound environments (18, 36). By impacting multiple stages of wound healing and targeting upstream actors in cell proliferation, polarization, and differentiation, MSCs can arguably offer wider and better therapeutic potential compared to existing diabetic wound treatments.

### 1.3 MSC use in diabetic cardiovascular disease

Cardiovascular diseases remain the leading cause of death in individuals with diabetes (1). Extensive research has shown that acute hyperglycemic states, as seen in diabetic patients, lead to elevated levels of nitrotyrosine, a reactive nitrogen species indicative of cell damage and inflammatory responses (38). Chronic hyperglycemia can result in prolonged inflammation and exacerbate the pathogenesis of cardiovascular diseases. Patients with T1D experienced prolonged QTc intervals compared to healthy controls and therefore were at greater risk for cardiovascular events (39). Understanding the intricate molecular mechanisms governing this relationship is imperative in creating targeted therapeutics aimed at mitigating the cardiovascular complications associated with diabetes.

One pivotal pathway under investigation is the phosphatidylinositol-3-kinase (PI3K) pathway. Phosphatidylinositol-3-kinases are a family of G-protein coupled receptors involved in cell proliferation, glucose transport, and inflammation (40). Specifically, the PI3K pathway is linked to the activation of lymphocytes and is therefore implicated in many inflammatory and autoimmune diseases (41).

Given the link between PI3K, inflammation, and diabetes, there is growing curiosity about how PI3K modulation could offer potential therapeutic benefits in addressing inflammatory-mediated diabetic heart disease. It has been demonstrated that diminished expression of the P110α isoform of PI3K (PI3K(p110 α)) correlates with heightened oxidative damage and worsened development of diabetic cardiomyopathy in murine models of T1D (42). Additionally, inhibition of PI3K(p110 α) impedes insulin signaling in the liver, resulting in diminished glucose uptake, impaired glucose metabolism, and overall increased hyperglycemia (43). Mice with T1D treated with a single-dose of PI3K(p110 α) experienced lower levels of oxidative reactants (43). These studies not only suggest that a deficiency in PI3K(p110 α) contributes to the pathogenesis of diabetic-associated heart disease, but also that PI3K(p110 α) is a potential therapeutic target in treating diabetic cardiovascular complications.

In targeting the PI3K pathway, MSCs hold immense therapeutic potential. MSCs can activate the PI3K pathway and exhibit antioxidant effects (44). For a diabetic patient, this holds potential to not only prevent the exacerbation of cardiovascular disease but possibly exhibit cardioprotective effects as well. Using MSCs as a method for enhancing PI3K(p110α) activity within the diabetic heart presents a promising strategy to counteract the adverse remodeling and dysfunction characteristic of diabetic heart disease. Through targeted delivery, MSCs could enhance PI3K(p110α) signaling specifically within cardiac tissue, promoting cardiomyocyte survival, attenuating fibrosis, and mitigating oxidative stress.

## 2 Discussion

The use of MSCs for complications of T1D is a promising avenue for the future. Through preclinical studies in animal models, MSCs have been shown to reduce the presence of pro-inflammatory cytokines and alleviate the potential for fibrosis and mesangial cell thickening often associated with diabetic nephropathy. They have demonstrated promise in enhancing tissue remodeling and angiogenesis in diabetic wounds. MSCs could additionally protect against diabetes-induced cardiovascular diseases by potentiating the PI3K pathway.

MSCs can address multiple diabetic issues through similar pathways and actors, highlighting the potential for combinatory effects. **Figure 2** demonstrates the pathways involved in the development of the various complications discussed here, and highlights where MSCs may modulate these pathways. In treating diabetic wounds, the effects of MSCs on the PI3K/threonine pathway are implicated in improving inflammatory stages and promoting angiogenesis. Similarly, in diabetic cardiac issues, the inflammatory pathway involving PI3K can be improved with MSC therapy. Likewise, the effect of MSCs in lowering inflammation by altering the activity of macrophages is seen in the prevention of nephron damage where MSCs lower macrophage infiltration, and in diabetic wound healing where MSCs polarize inflammatory M1 macrophages to anti-inflammatory M2 macrophages. Overall, MSC therapy could revolutionize the management of T1D complications through anti-inflammatory regulation, tissue regeneration, and pathway modulation.

While research on animal models has demonstrated benefits for diabetic wound treatment, clinical work has highlighted the practical therapeutic potential of MSCs while also raising concerns for adverse effects. Umbilical cord MSCs can increase and renew vasculature and improve the completion of diabetic wound healing through intravascular and intralesional injections (23). Similarly, it was demonstrated in a cohort of 59 patients that adipose-derived MSCs can quicken the rate of wound healing and the time it takes for a lesion to close (21). While this clinical work highlights the immense benefit of MSCs towards diabetic wounds, adverse clinical outcomes including febrile neutropenia, alopecia, gastrointestinal reactions, and relapse of diabetic injury in patients with T1D introduce controversy as to how and if MSC therapy for diabetic wound should be applied (22, 45). Investigating how MSCs can be combined gradually with existing wound treatment techniques and preventative measures against their anticipated adverse effects may provide safer pathways to applying MSC therapy without ignoring the transformative potential MSCs provide to diabetic individuals’ quality of life.

In the case of diabetic cardiovascular diseases, its application of the PI3K pathway in the context of T1D remains relatively unexplored. Most current research on PI3K modulation focuses on type 2 diabetes (T2D), where insulin resistance plays a central role in the pathophysiology of the disease. However, T1D, characterized by autoimmune destruction of pancreatic beta cells and insulin deficiency, presents distinct challenges and mechanisms. One complication of this discrepancy is that the potential therapeutic benefits of PI3K using MSCs may not be fully applicable to T1D. The underlying pathogenesis and disease mechanisms differ significantly between T1D and T2D, potentially affecting the efficacy of treatment strategies (1). Moreover, the limited knowledge and current research available on PI3K modulation of T1D further complicates efforts to translate findings from T2D to T1D.

The appeal of MSC therapy, however, lies in its potential advantages over conventional treatments for diabetes (46). Currently, insulin-based remedies remain the mainstay for management in T1D patients (1). If a patient presents with diabetic complications, additional symptom-based treatments are necessary as well. Over time, these therapies become financially demanding, resulting in many patients forgoing essential medications. While the initial investment in MSC research and therapy development may be high, the long-term benefits of an upfront MSC therapy could outweigh the expenses associated with ongoing treatments.

Moving forward, additional research is needed to bridge the gap between preclinical studies and clinical trials. While producing functioning beta cells *in vitro* and in animal models has been successful, similar success has been promising but rare in human patients (47). **Table 2** offers a synopsis of active, completed, and terminated clinical trials investigating the use of MSCs in the treatment of T1D. While the number of ongoing studies and growing interest in this field is exciting, no clinical trial has yet reached Phase III or further, and none have demonstrated sustained effects in human participants so far. The relative lack of treatment success in humans to date highlights the need for further studies to better understand the discrepancy between the responses seen in animal models versus humans. Critical questions also remain unanswered regarding the long-term effects of MSC treatments, including the possibility of adverse immune reactions, tumorigenesis, and unintended consequences depending on individual physiology. Moreover, the variability in MSC sources, methods of isolation, and delivery techniques further emphasizes the need for standardized protocols and comprehensive safety assessments.

**Table 2:** Summary of active, completed, and terminated clinical trials investigating MSCs for the treatment of T1D.

NCT Number	Official Title	Study Start Date	Status	Phase	Purpose of the Study	Treatment Method
NCT06407297	Investigation of Dose Escalation and Cohort Expansion Study on the Safety and Efficacy of Allogeneic Umbilical Cord Mesenchymal Stem Cells(UCMSCs) Combined With Standard Therapy for Newly Diagnosed Type 1 Diabetes	2024-05-23	Recruiting	Not Applicable	Investigate the safety and tolerability of intravenous infusion of allogeneic umbilical cord MSCs in pediatric patients diagnosed with newly onset T1D	Peripheral intravenous infusion of allogeneic umbilical cord MSCs
NCT05061030	A Double-blinded, Randomized, Parallel, Placebo-controlled Trial of Wharton’s Jelly-derived Allogeneic Mesenchymal Stromal Cells to Treat Type 1 Diabetes in Children and Adolescents	2022-01-14	Recruiting	Phase I/II	Investigate the safety, tolerance, and efficacy of an allogeneic infusion of Wharton’s jelly derived MSCs	Intravenous infusion of allogeneic Wharton’s jelly-derived MSCs (ProTrans)
NCT04061746	Cellular Therapy for Type 1 Diabetes Using Mesenchymal Stem Cells	2020-02-27	Recruiting	Phase I	Determine the safety and efficacy of allogeneic umbilical cord-derived MSCs for the treatment of new-onset T1D, understand the mechanisms of protection	Infusion of allogeneic umbilical cord-derived MSCs
NCT03973827	An Open Label, Parallel Single Centre Trial of Wharton’s Jelly Derived Allogeneic Mesenchymal Stromal Cells Repeated Treatment to Preserve Endogenous Insulin Production in Adult Patients Diagnosed with Type 1 Diabetes	2019-05-17	Completed, 2024-11-20	Phase I/II	Determine whether a repeated allogeneic infusion of WJMSCs in adult T1D patients is safe; study changes in beta-cell function, metabolic control and Diabetes Treatment Satisfaction	Allogeneic transplantation of Wharton’s jelly-derived MSCs (ProTrans)
NCT06812637	Efficacy and Safety of Wharton’s Jelly-Derived Mesenchymal Stem Cell Exosomes in the Treatment of Diabetic Foot Ulcers: a Randomized Controlled Trial	2024-04-06	Completed, 2024-09-02	Phase I	Investigate the efficacy and safety of the topical application of Wharton’s jelly derived MSC exosomes in patients with chronic diabetic foot ulcers	Standard of care with Wharton’s jelly-derived MSC exosome gel once weekly for 4 weeks
NCT03920397	Allogeneic Adipose Derived Mesenchymal Stem Cells and Vitamin D Supplementation in Patients With Recent-onset Type 1 Diabetes Mellitus	2015-03-01	Completed, 2021-05-01	Not Applicable	Test the efficacy of an intravenous infusion of adipose tissue-derived stromal cells in patients with recent-onset T1D	Infusion of adipose tissue-derived stem/stromal cells and oral Cholecalciferol supplementation
NCT03406585	A Double-blinded, Randomized, Placebo-controlled Trial With Wharton’s Jelly Derived Allogeneic Mesenchymal Stromal Cells (WJMSCs) for Preserving Endogenous Insulin Production in Adult Patients Diagnosed for Type 1 Diabetes	2017-11-28	Completed, 2020-09-04	Phase I/II	Investigate the safety and tolerance after allogeneic infusion of Wharton’s Jelly derived MSCs intravenously in adult T1D patients	Infusion of a cell suspension with allogeneic MSCs derived from umbilical cord tissue
NCT01068951	Open Study to Evaluate the Safety and Efficacy of Autologous Mesenchymal Stem Cells in Treatment of Recently Diagnosed Patients With Type 1 Diabetes Mellitus	2010-06	Completed, 2013-09	Not Applicable	Evaluate the safety and efficacy of autologous MSCs in recently diagnosed patients with T1D	Intravenous autologous transplantation of the patients’ own MSCs
NCT00690066	A Phase II, Multicenter, Randomized, Double-Blind, Placebo-Controlled Study to Evaluate the Safety and Efficacy of PROCHYMAL^®^ (Ex Vivo Cultured Adult Human Mesenchymal Stem Cells) for the Treatment of Recently Diagnosed Type 1 Diabetes Mellitus	2008-06-11	Completed, 2011-12-19	Phase II	Establish the safety and efficacy of multiple administrations of PROCHYMAL^®^ in participants recently diagnosed with T1D	Intravenous infusion of ex vivo cultured adult human MSCs (PROCHYMAL^®^)
NCT03840343	Intra-arterially Delivered Autologous Mesenchymal Stem/Stromal Cell Therapy in Patients With Diabetic Kidney Disease: A Phase I Study	2019-10-23	Terminated, 2020-08-04	Phase I	Assess the safety, tolerability, dosing effect, and efficacy of intra-arterially delivered autologous adipose tissue-derived MSCs in patients with progressive diabetic kidney disease	Lower Dose: Two intra-arterial MSC infusions of 2.5x10^5 cells/kg 3 months apart Higher Dose: Two intra-arterial MSC infusions of 5.0x10^5 cells/kg 3 months apart

Clinical trials were identified from the clinicaltrials.gov database using the search tool criteria “Type 1 Diabetes” or “Type 1 Diabetes Mellitus” (Condition/disease), and “Mesenchymal Stromal Cells” or “Mesenchymal Stem Cells” (Intervention/treatment).

Questions also remain regarding personalized treatment approaches, considering T1D may present in a variety of ways. Further research must also be done on the long-term implications of prolonged MSC therapy, and strategies to mitigate potential adverse reactions. Despite these remaining challenges, MSC therapy holds potential for improved patient outcomes through an upstream model for T1D complications that could enhance patient accessibility and treatment adherence. Currently, conventional insulin treatments require daily injections and lifelong monitoring. The less frequent use of MSC treatments could improve treatment adherence and relieve the mental fatigue that comes with daily treatments. Considering the success of MSC therapy in other medical domains such as cancer treatment, there is promising potential to advance MSC therapy for diabetes with ongoing research and innovation.
